# Solutions for improved hospital-wide patient flows – a qualitative interview study of leading healthcare providers

**DOI:** 10.1186/s12913-022-09015-w

**Published:** 2023-01-07

**Authors:** Philip Åhlin, Peter Almström, Carl Wänström

**Affiliations:** Department of Technology Management and Economics, Chalmers University of Technology, Vera Sandbergs Allé 8, 412 96 Göteborg, Sweden

**Keywords:** Healthcare, Efficiency, Productivity, Process improvements, Organizational efficiency, Capacity utilization, Strategy, Hospital ranking

## Abstract

**Background:**

Hospital productivity is of great importance for patients and public health to achieve better availability and health outcomes. Previous research demonstrates that improvements can be reached by directing more attention to the flow of patients. There is a significant body of literature on how to improve patient flows, but these research projects rarely encompass complete hospitals. Therefore, through interviews with senior managers at the world’s leading hospitals, this study aims to identify effective solutions to enable swift patient flows across hospitals and develop a framework to guide improvements in hospital-wide patient flows.

**Methods:**

This study drew on qualitative data from interviews with 33 senior managers at 18 of the world’s 25 leading hospitals, spread across nine countries. The interviews were conducted between June 2021 and November 2021 and transcribed verbatim. A thematic analysis followed, based on inductive reasoning to identify meaningful subjects and themes.

**Results:**

We have identified 50 solutions to efficient hospital-wide patient flows. They describe the importance for hospitals to align the organization; build a coordination and transfer structure; ensure physical capacity capabilities; develop standards, checklists, and routines; invest in digital and analytical tools; improve the management of operations; optimize capacity utilization and occupancy rates; and seek external solutions and policy changes. This study also presents a patient flow improvement framework to be used by healthcare managers, commissioners, and decision-makers when designing strategies to improve the delivery of healthcare services to meet the needs of patients.

**Conclusions:**

Hospitals must invest in new capabilities and technologies, implement new working methods, and build a patient flow-focused culture. It is also important to strategically look at the patient’s whole trajectory of care as one unified flow that must be aligned and integrated between and across all actors, internally and externally. Hospitals need to both proactively and reactively optimize their capacity use around the patient flow to provide care for as many patients as possible and to spread the burden evenly across the organization.

**Supplementary Information:**

The online version contains supplementary material available at 10.1186/s12913-022-09015-w.

## Background

Demand for healthcare is rising faster than available capacity and is considered to be caused by changing demographics and increasing multi-morbidity [1, 2] in combination with chronic healthcare staffing shortages [3–7]. Simultaneously, healthcare systems annually acquire larger portions of the national GDP, reducing the will of policymakers to continuously inject the financial support the sector requests [8–11]. Altogether, this causes prolonged waiting times for care, and the health sector’s possibility to treat patients at the right time with the level of care they need is reduced [3, 7, 12, 13]. The need for improvement is urgent, especially in hospitals, the largest receiver of healthcare funding [14, 15]. Therefore, healthcare managers must look for new solutions to improve hospitals’ capacity utilization to increase productivity without further increasing expenditures. The last two decades have seen a growing interest in how to improve healthcare productivity by focusing more on the patient flow, i.e. how to enable a higher throughput of patients through hospitals [12, 16–22]. Focusing on the flow of patients has been proven to decrease patients’ length of stay (LoS) and increase the speed with which patients are processed toward discharge [13, 20, 23–25]. It may also help balance a varying number of patients along a continuum of care constrained by insufficient healthcare resources [22]. Additionally, a long LoS exposes patients to unnecessary risks of iatrogenic complications such as infections [21]. A greater focus on the patient flow is therefore recognized as critical to improve not only productivity but also medical quality, patient safety, and patient satisfaction [25, 26].

Many years back, Vissers et al. [27] and Litvak and Bisognano [28] highlighted the importance of using a system-wide lens when improving patient flows across hospitals. This perspective emphasizes the consideration of problems or bottlenecks associated with the flow of patients along a continuum of care throughout the organization [29]. It highlights that a plethora of clinics and medical units within hospitals, caring for the patient between admission and discharge, must align their objectives to make the hospital efficient and effective in delivering the right care at the right time and place and at the right cost [21, 22]. However, today this system-wide approach to patient flow is still mostly used rather superficially to denote merely that flow improvement requires intervention in more than one part of the system [29]. One explanation comes from the hardship healthcare managers face in employing a hospital-wide perspective on patient flows, as hospitals are internally divided, with departments and clinics not sharing the same objectives and often competing over common resources and the availability of various services [30–32]. Hence, studies on how to improve patient flow rarely encompass complete patient processes throughout the hospital, from admission until discharge [32, 33]. Instead, the focus is most often narrower, looking at the patient flow through single clinics or units [12, 22, 33]. In this light, recent research expresses the need for more studies on prescriptions to actually improve system-wide patient flows within hospitals. The research emphasizes the need for more evidence-based studies that can provide better guidelines to handle the contextual and causal complexities of the hospital associated with improving hospital-wide patient flows [12, 20, 22, 34, 35].

The aim of this study is, therefore to (i) identify effective solutions to achieve swift patient flows across hospital organizations and (ii) develop a framework to guide improvements in hospital-wide patient flows.

To address this aim, we have conducted an international interview study with senior managers at 18 large academic hospitals to explore how they perceive patient flows from a system-wide perspective and to understand their strategies on how to improve the flow across their organizations. Hospitals are acknowledged as highly complex organizations comprising strong professional groups with oftentimes different views on improving the healthcare sector [31, 32, 36]. Process improvement models originating from the industrial environment are therefore seldom easy to implement in healthcare organizations [32, 33]. Leading academic hospitals encompass the height of complexity within the healthcare sector, considering the significant number, variety, and complexity of patients they treat while fulfilling large teaching and research requirements. Consequently, they most likely face more obstacles and challenges compared to other hospitals when trying to improve their processes. Academic hospitals also generally achieve higher medical performance than other hospitals [37–39], presumably supported by leading practice in flow logistics, a connection found in previous research [25, 26]. The external requirements on these care providers to deliver high performance are also significant, as providers receive considerable funding from governments and public institutions for their research and teaching programs. Consequently, their solutions to swift hospital-wide patient flow should not only be specifically interesting but likely applicable to a wider range of other hospitals with less complex organizational structures. Moreover, representatives with a good understanding of the complete organization of the hospital and the various improvement projects conducted across the hospital are generally senior managers. They may not provide the same in-depth understanding as a large group of physicians or nurses spread across a healthcare organization. However, they do possess a holistic view of the problems facing hospitals and have relevant perspectives (from strategic to operative) when discussions are held at a more general level. Hence, senior managers at leading academic hospitals serve as study objects in this interview study on solutions to swift hospital-wide patient flows.

**Fig. 1:**
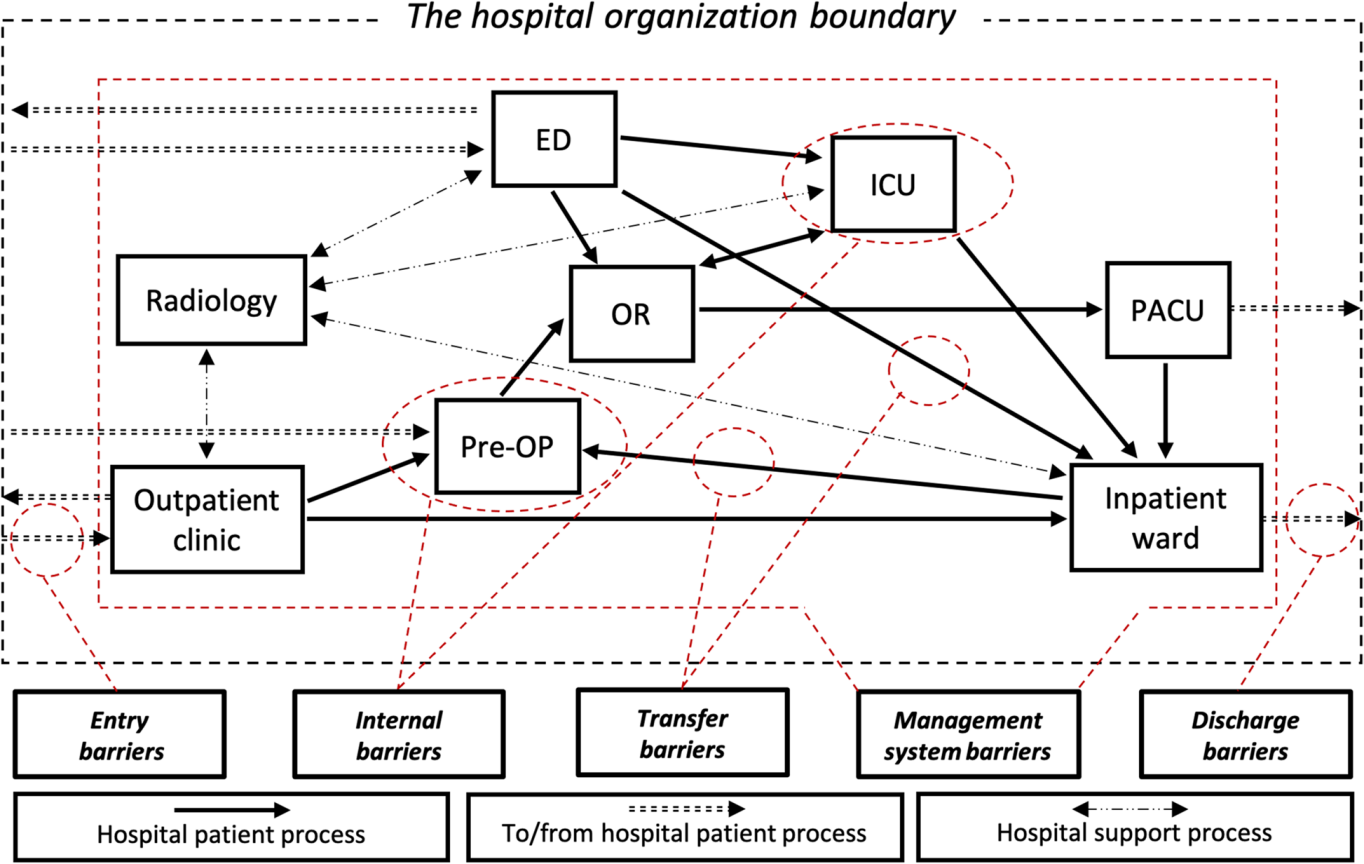
The hospital-wide process model, Åhlin et al., [40]

This paper builds on our previous study [40], a systematic literature review on what is preventing swift hospital-wide patent flows. According to Devaraj et al. [21], it is necessary to understand the constraints behind processes before trying to improve them. They point to the need to identify and describe the bottlenecks in a system before breaking them [41, 42], something further articulated by the law of bottlenecks, which states that the overall efficiency of a process can only be improved by addressing its major bottlenecks or constraints [29, 42, 43]. Consequently, based on the categories of processes presented by Holweg et al. [41], we developed a hospital-wide process model depicting five general themes of barriers patients moving through a hospital organization may face [40]. These are: Entry (the entry of patients to the hospital organization); Transfer (the movement of patients between clinics or departments); Internal (the treatment of patients within clinics or departments); Management system (the system-wide planning and control of the patient flow through the hospital); and Discharge (the exit of patients from the hospital organization), see Fig. 1. The model visualizes the patient process from admission to discharge through the central settings of a hospital organization, the patient processes to and from the hospital, and the supporting processes.

From our previous study, we have also developed a framework for what prevents the achievement of efficient patient flow across hospital organizations [40]. This framework describes 12 main barriers and 15 main root causes of inefficient patient flow categorized under the five previously described themes of barriers; see Table 1. This framework acts as a starting point, and in this study, we connect barriers with solutions to provide healthcare managers, commissioners, and decision-makers with an extended framework consisting of both barriers and solutions to swift hospital-wide patient flows.

**Table 1:**
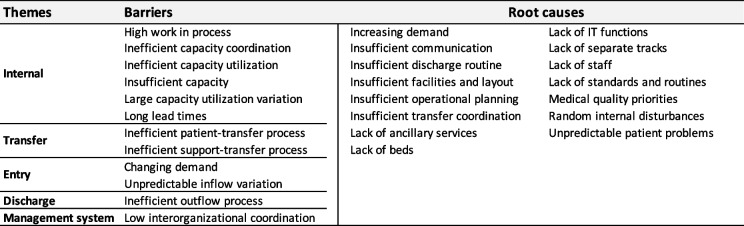
Themes, barriers, and root causes of inefficient patient processes

## Methods

### Design

We have taken an explorative qualitative approach throughout this study, encompassing both deductive and inductive elements. A deductive methodological framing has been used, taking previous research as a starting point to extend a framework for efficient hospital-wide patient flows, presented by Åhlin et al. [40], with new perspectives. The framework has shaped the data collection method and understanding among researchers of the problems the study objects face. A thematic analysis of the collected data has, however, also been conducted with a clear inductive research approach, chosen to carefully explore the subjective views of the study objects, as suggested by Braun and Clarke [44] and Dixon-Woods et al. [45]. This has been chosen instead of, based on prior research, looking for particular categories associated with the framework in a more deductive manner. Lastly, evolving themes from the thematic analysis were related back to the framework.

### Data collection

In-depth, semi-structured interviews were used as a primary data source and together with the use of an interview guide, see Additional file 1: Appendix A, we ensured both comparability between interviews and openness to new ideas and perspectives [46]. Questions that guided the data collection throughout each interview were structured one by one to subsequently seek answers on how to best overcome each barrier described in the framework. When finding suitable participants, we used the 2020 international hospital ranking by the American magazine *Newsweek* [39], which presents an annual list of hospitals and medical clinics around the world based on recommendations from medical experts (doctors, hospital managers, healthcare professionals), results from patient surveys, and central medical KPIs. An initial inquiry was sent to the president or CEO of each hospital organization, whereupon the inquiry was often, but not always, forwarded to another manager better suited to answer our questions. If willing to participate, an online meeting was scheduled, and if the hospital found it appropriate, more than one participant took part in the interview. Consequently, the role of the participants varied slightly from the CEO or the president to the chief operating officer or the hospital medical director, and in a few cases, the flow department manager was interviewed. The selection criteria for participation were: senior managers with (i) responsibility for patient flow-related questions and (ii) a responsibility covering the whole or, at least, large parts of the hospital organization. Most participants had a professional background as physicians, a few were nurses, and a small number had a non-care related background. To improve the validity of the study, a pilot study was conducted with three regional hospitals in Sweden, whereafter each interview was transcribed verbatim and analyzed. The outcome slightly changed the interview guide and gave a better understanding of how to balance time between questions and formulate follow-up questions. Following the pilot study, the inquiry was sent to the 25 highest-ranked hospitals according to the list by *Newsweek* [39], and 18 hospitals accepted the invitation leading to a first interview, see Table 2.

**Table 2:**
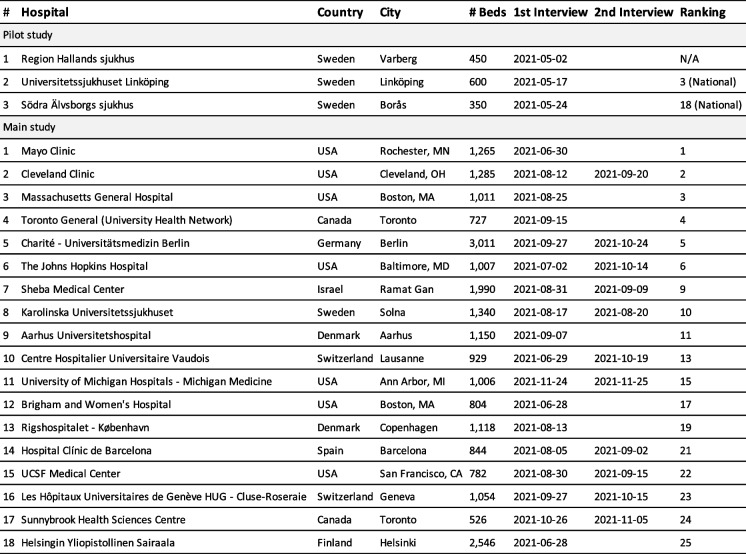
Interview study participant list

Interviews were held with one or two managers, followed by a second interview when needed with either the same person(s) or another manager. Thirty interviews were held with, in total, 33 hospital managers, and only one manager later decided to withdraw participation, based on a retrospective judgement of not being the most suitable person to answer the questions. The interview guide was sent to every participant ahead of the interview, whereupon interviews were carried out by one of the authors (PÅ) between June 2021 and November 2021. Following the interview guide, each participant was asked what they and their hospital do to subsequently overcome each patient flow barrier. The main role of the interviewer was to enable an open and friendly format, introduce each subject, and then follow up actively with requests for further elaboration and clarification. The interviews lasted between 60 and 90 min, whereupon 11 of the interviews had to be extended with a complimentary session to get other person’s views and to ensure that all questions were answered. The participants had different backgrounds and pre-understanding of the concepts discussed; therefore, some needed more prompting than others to appreciate all questions. All interviews were conducted online, using the online meeting software Zoom, as the COVID-19 pandemic prevented physical meetings.

### Data analysis

All interviews were recorded and transcribed verbatim and sent back to respondents for approval. All authors read through and familiarized themselves with the transcriptions before thematizing the content to obtain a sense of the whole. One of the authors (PÅ) open-coded the verbatim transcripts, seeking to capture all expressed opinions and recommendations, enabling a vast number of unique aspects; see Additional file 2: Appendix B. Each aspect expressed a “solution” on how to overcome a particular barrier, and was consequently mapped to that barrier. This resulted in a large number of opinions and recommendations associated with at least one barrier of the framework. In a few cases, interviewees gave perspectives that did not address any pre-existing barrier of the framework. Hence, based on the interview material and discussions between (PÅ), (PA), and (CW), new barriers had to be constructed, whereupon these solutions were matched with the newly developed barriers. Following this, all open codes of solutions associated with one or several barrier(s) were discussed among all three researchers for aggregation into themes and higher levels of abstraction. This thematization ended when data saturation had been reached, no more solution categories could be identified, and consensus had been reached among the researchers.

## Results

We present our findings from an overall observational perspective as well as from a deeper level with explanations of the underlying structures, supported by representative quotes from the interviews. The interviews yielded 558 unique opinions and recommendations, resulting in 50 solutions presented and indexed in Table 3. The right column in the table presents all solutions, and the middle column presents the barriers the solutions help to overcome. Lastly, the left column presents themes of barriers to visualize where along the patient flow these barriers evolve and, consequently, where the identified solutions provide support.

**Table 3:**
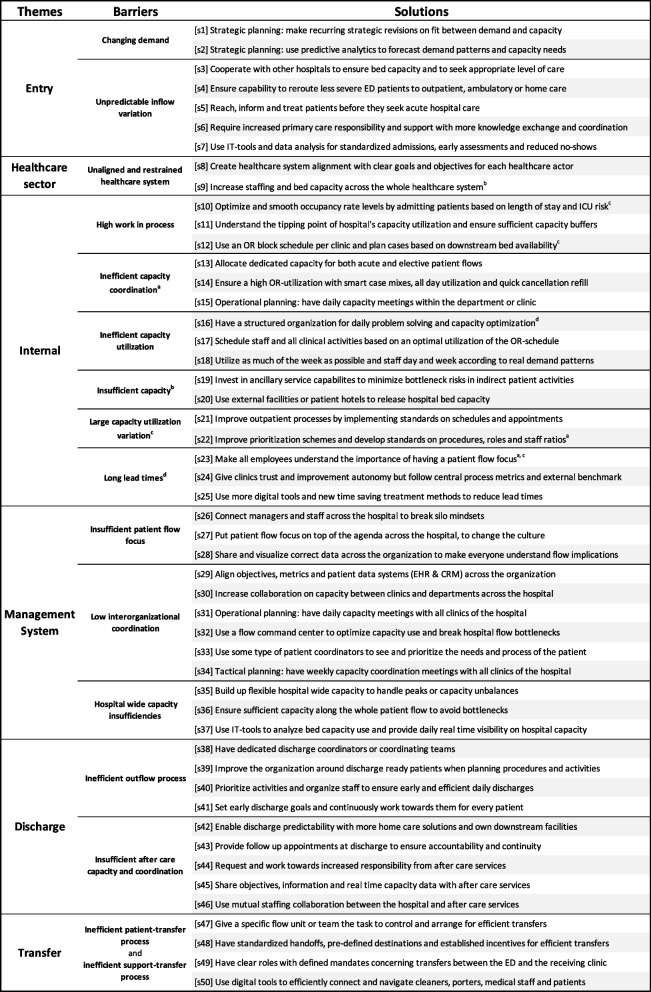
Themes, barriers, and solutions to efficient patient flows

^a^^, b, c, d^refers to a connection between a solution and more than one barrier

To a large degree, Table 3 gives a unified picture among the hospitals of what challenges they meet and what solutions they seek or prioritize. On average, nine hospitals support or prioritize each solution; see Additional file 3: Appendix C. In Additional file 3: Appendix C, it is also possible to see that the prioritized solutions are evenly spread along the hospital patient process, addressing all themes of patient flow.

Beyond previously identified themes and barriers [40], one new theme and four new barriers were developed. We introduced the theme “*Healthcare sector”* together with the barrier “*Unaligned and restrained healthcare system”,* as multiple hospitals point to problems in aligning different healthcare providers and to staff and bed insufficiencies across the healthcare system. Two barriers were introduced under the theme “*Management system*” to emphasize problems with a hospital culture not directed towards a flow perspective: *“Insufficient patient flow focus”,* and problems associated with capacity insufficiencies hurting the whole hospital and not just single clinics: “*Hospital-wide capacity insufficiencies”.* Lastly, the barrier “*Insufficient aftercare capacity and coordination”* was introduced, as multiple problems are associated with the transfer of patients and the coordination and cooperation with aftercare services. This addition fills a gap, as few barriers have previously been found associated with the management system; the systematic review by Åhlin et al. [40] mainly included empirical studies of improvement projects in single settings. All themes, barriers, and solutions are presented in Table 3, with rows representing their connections. Superscripts in Table 3 indicate that one particular solution has a connection to more than the nearest barrier, within the same row.

The 50 solutions are explained below, together with representative quotes from the interviews under eight summarizing categories: 1. *Align the organization* describes the need to work towards a unified goal with a unified strategy throughout the whole organization; 2. *Build a coordination and transfer structure* describes the need to ensure quick and precise communication supported by clear mandates along the whole patient flow, from primary care to aftercare services; 3. *Ensure physical capacity capabilities* describes the need to create flexibility by investing in important spaces and places to enable greater buffer systems and peak census management; 4. *Develop standards, checklists, and routines* describes the need to make processes more clear and foreseeable to both patients and practitioners, as well as administrators; 5. *Invest in digital and analytical tools* describes the need to use available modern and smart IT services for quicker and better decisions; 6. *Improve the management of operations* describes the need to continuously assess capacity and optimize operations, both centrally and locally, to dissolve patient flow bottlenecks; 7. *Optimize capacity utilization and occupancy rates* describes the need to proactively plan activities to smooth resource utilization across the whole organization to make the organization more balanced and efficient; 8. *Seek external solutions and policy changes* describes the need to work towards a better-staffed, more patient-centric, and more aligned healthcare sector. To facilitate the reading, since the number of solutions is considerable, Table 4 visualizes the category in which each solution is presented.

**Table 4:**
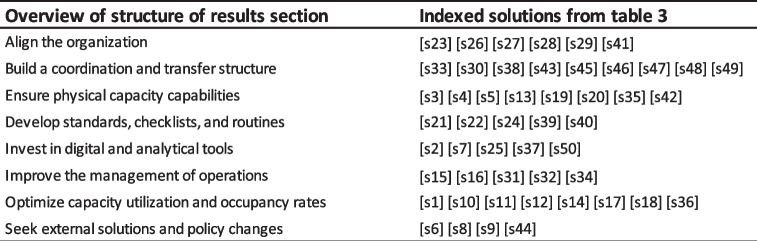
Overview of the results section

### Align the organization

Planning for an efficient flow along a patient’s whole trajectory of care involves the need to approach heterogeneous clinical conditions, varying practices, routines, competing organizational objectives, and multiple local cultures. Therefore, the solutions [s23, s26, s27, s28, s29, s41] highlight the need to align the organization and address these challenges to improve the flow of patients. Several hospitals already consider it important to try to estimate the day of discharge upon patient admission and, if possible, have the entire organization focus on reaching that goal [s41]. To continuously improve this practice, statistical feedback loops are required to ensure precise estimations and enable root cause analyses behind potential deviations. This must also be supported by aligning the organization’s objectives, metrics, and data systems to ensure that everyone shares the same view along a continuum of care [s29]. A patient flow focus within each clinic [s23] and across the hospital is important [s27], emphasizing the need for everyone to understand implications along the flow and take responsibility for the consequences of certain decisions, as described by this medical officer:

*“If, for example, neurosurgeons have the goal to run neurosurgical care to the right quality and resource, then they are responsible for the final quality, waiting times, and costs along the entire flow until that patient is discharged. It is then likely that the heads of neurosurgery will need to spend 80 to 90 percent of their working time on activities outside of neurosurgery to strengthen them if they work poorly.” -* Medical Director, Karolinska Universitetssjukhuset

Focusing on the patient flow highlights the importance of employees seeing the needs of the hospital and the whole population of patients along the patient flow before the needs of their own clinic or unit. Managers must also be committed to this change by building relationships and seeking cooperation across departmental borders to break silo mindsets [s26]. An open and collaborative culture must also build on shared visibility and transparency through correct data analyses [s28], as seen here:

*“There are cultural difficulties, but if you can bring the data and show the correct numbers, things can improve. Before, people said, ‘you do not have the right data because you say that I have six patients, but I have seven’. Shared visibility of what's really going on gives solidarity between departments like ‘Oh, last weekend it was terrible for you, so I will give you some resources to cope with this influx of patients’. This is how you change the culture.” -* Deputy Director General, Les Hôpitaux Universitaires de Genève

### Build a coordination and transfer structure

Along the complex chain of events making up the patient flow, good coordination is needed between internal and external actors to align activities, handoffs, and transfers of patients [s33, s30, s38, s43, s45, s46, s47, s48, s49]. Efficient transfers between the ED and inpatient wards are supported by having specific flow units or teams in charge of both transfers and assignments of beds [s47]. Clear roles for everyone involved in transfer arrangements are also important, where the person or team in charge of transfers needs a strong mandate [s49]. Moreover, standardized handoffs, pre-defined destinations for patients with certain diagnoses, and clear incentives to promote efficient transfers are needed [s48], as highlighted by this senior vice president:

*“We start to standardize the communication in referral pathways so that handoffs actually contain relevant clinical issues. It helps to prioritize how patients are admitted and to what services, so we don't spend as much time figuring out which services patients go to. Internally, we have also created a detailed admission document so that there's no delay once a clinical reason for admission has been established. It's all predetermined.” -* Senior Vice President, Massachusetts General Hospital

Several hospitals emphasize the benefit of having specific patient coordinators to see the need of patients and plan care pathways to make them smooth and coordinated [s33]. It is also important to have case managers, social workers, coordination consultants, or discharge teams to proactively plan for a seamless and well-organized discharge process [s38]. Concerning the flow out from the hospital, a strong collaboration between hospitals and aftercare services and the presence of external staff to expedite the transfer of patients is recommended [s46]. Having nurses, physicians, and outgoing teams visit aftercare facilities also provides better alignment, and multiple hospitals point to the need to share objectives and information with aftercare services for better collaboration [s45]. Moreover, proactively planning for the continuous care of patients by providing follow-up appointments at discharge can further strengthen transfers and improve patient safety [s43]. Better coordination of patients and a stronger transfer structure are also supported by closer collaboration on the relocation of patients between departments and clinics [s30], exemplified by this medical officer:

*“Instead of each unit operating as a silo, we have units operating together as pools of capacity, a medicine pool, surgical pool, cardiovascular pool. Multiple units can take the same type of patient for a larger overall capacity. Obviously, you need the right staff and skill for this flexible model, but that has allowed us to be more nimble.” -* Associate Chief Medical Officer, University of Michigan Hospitals

### Ensure physical capacity capabilities

Acting in a highly varying and often unpredictable environment requires flexible capacity capabilities that provide sufficient margins to avoid bottlenecks associated with overcrowding [s3, s4, s5, s13, s19, s20, s35, s42]. Hospitals must be able to reroute patients to avoid acute hospitalization, and internal command centres or flow teams can then provide good support. Clearly defined pathways are needed from the ED to outpatient, ambulatory, or home care settings [s4] or to other hospitals or secondary care providers [s3]. Hospitals must also proactively reach, inform, and treat patients before they seek acute care by expanding the prehospital with mobile care teams, virtual EDs, 24–7 off-hours hotlines, and expanded telemedicine capacity [s5], as described by this general director:

*“We are improving the prehospital, the organization running the ambulances. They have a great impact on the treatment of patients as their doctors in passenger cars sometimes arrive before ambulances. When people call 112, the prehospital ends about 10 percent of all cases. We constantly aim higher and ask if the next level could be 20 percent?”-* CEO, Aarhus Universitethospital

Several hospitals emphasize the need to build flexible hospital-wide capacities like floating nursing pools to handle peaks in demand and sudden capacity imbalances pointing to interim personnel units, multi-speciality wards, and short-stay units (SSU) [s35]. External facilities such as discharge lounges, patient hotels, and specific facilities for long-term transplant patients are also promising solutions [s20]. Some hospitals acknowledge the benefit of ensuring sufficient ancillary services capacity to avoid bottlenecks in direct patient activities [s19]. Having separate operating theatres for acute and planned surgeries is also helpful [s13]. Concerning the flow out from the hospital, some hospitals might consider opening aftercare services to ensure downstream bed capacity. A more common solution is to invest in home-care solutions for higher discharge predictability and to release bed capacity. However, this raises questions on how and where to care for patients and must be arranged with primary care and aftercare services. Lastly, home care is supported by in-home monitoring and using outgoing home care teams [s42]. This is described by one manager:

*“We're opening a new telemedicine hospital called Sheba Beyond. So, if patients are not in critical care or prepared for the operation, they can go home. We can monitor them at home or at their nursing home. Our physicians, through telemedicine, can take care of the patient. The nursing home staff or the nursing staff from our hospital or the health care fund can go there and help, to prevent hospitalization. This is the new method we are working on. It is the future.” -* Associate Director General, Sheba Medical Center

### Develop standards, checklists, and routines

A myriad of activities is performed across the hospital every day by different people and in different manners. This creates significant internal variation and unpredictable patient processes. However, these can be mitigated by the introduction of more standards, prioritization schemes, and routines [s21, s22, s24, s39, s40]. Clarity of roles and procedures is emphasized concerning internal clinical activities like standards for nurse-bed ratios, work tasks, routes of communication, and decision-making [s22], as described by one medical director:

*“Healthcare largely ignores time. An expectation is set on how many patients to see, but we don't have a good sense of the time it takes to see those patients. We don't necessarily know and account for the time it is to check those patients in, schedule their follow-up visits, or to make calls to transfer them to another unit. Part of the capacity problem is because we've ignored time. We need to get better visibility to that. How long does each step take and then staff to that.”-* Chief Improvement Officer, Cleveland Clinic

Concerning clinical efficiency, several hospitals find it important to let clinics independently set goals and conduct improvement activities but then centrally follow up on performance measurements like the length of stay and bed and appointment utilization. Clinics are also compared against national benchmarks and internal capacity standards [s24]. Improving the processes of the outpatient clinics is also important, and standards are needed for schedules, clinical slots, and physician time [s21], as seen below:

*“There is great variability in the ambulatory clinics' capacity utilization, depending primarily on the scheduling slots, both in time duration and numbers. Three years ago, we started a pilot project where everyone in a particular division had to agree upon and standardize their clinical slots, the length [of] time for each one, and start and end time. A part of it was that we wanted to go to electronic self-scheduling so that patients can schedule themselves.” -* Interim President, Brigham and Women's Hospital

To improve discharge procedures, hospitals point to the need to introduce clear daily routines and prioritization schemes to ensure all necessary activities are synchronized and finished in time for early discharges [s40]. This must be supported by an organization-wide prioritization of the last steps for discharge-ready patients and that physicians prioritize discharge-ready patients more in relation to other activities [s39].

### Invest in digital and analytical tools

Hospitals are complex, and acquiring a holistic view of the organization and its processes is hard. Therefore, a focus on digital and analytical tools is increasingly emphasized for better and informed decisions and to provide technical support around the flow of patients [s2, s7, s25, s37, s50]. Moreover, hospitals are investing in predictive analytics to anticipate demand patterns, future needs for beds and staff, arising bottlenecks, organizational risks, and scenarios following strategic decisions [s2]. This is described by one medical officer:

*“We've created an IT tool that takes our scheduled activity, translates it into a calendar, giving us visibility to the anticipated bed use each day, using modelling of length of stay profiles. We know tomorrow there's cardiac surgery patients using four ICU beds, based on historical use. The combined cardiac surgery service will use 30, and then we can say it's going to be these many future bed days. We then translate it into anticipated occupancy to see the consequences on ED boarding.” -* Associate Chief Medical Officer, University of Michigan Hospitals

Concerning planned admissions of patients, digital tools become increasingly important when providing early video assessments or using robots and algorithms for automatic reading and sorting of referrals. Data analytics can also help standardise admission routines and reduce practice variability among physicians [s7]. Concerning capacity utilization across the hospital, demand heat mapping can be used to optimize the allocation of capacity, and real-time dashboards with relevant metrics improve the performance and control of operations [s37]. New technology can help digitalize radiology services and make patient flows and pathways more visual and transparent to both patients and staff [s25]. New IT systems can also quickly connect and direct providers and expedite the patient flow across the hospital [s50], as highlighted here:

*“When we transfer patients, we visit our service platform and move them with one click from where they are to where they are going to be transferred. We then place an order in our control system for a porter to move the patient physically. Since we started to connect the system with the porters’ telephones, they get quick information on what patient to bring where and at what time. It has immediately worked and been a real success.” -* CEO, Rigshospitalet København

### Improve the management of operations

How operations are executed requires good organization and efficient decision structures supported by clear communication channels to effectively manage available capacity [s15, s16, s31, s32, s34]. Multiple hospitals highlight a need for command centres to track and optimize daily capacity and to identify and act on arising bottlenecks [s32], as exemplified by one medical director:

*“The concept behind the command centres is that we're trying to put all individuals responsible for the hospital operations in the same room, looking at the same data at the same time. We're using dashboards within our electronic health record to tell us in real-time what the situation is like in the ED, in the hospital, in the OR, and on the various floors. The data produced in our command center is then used as a template for our daily morning huddles and is driving decision making regarding where patients might go throughout our system.” -* Hospital Medical Director, Mayo Clinic

To support command centres, it is important to have daily capacity meetings on anticipated admissions and discharges in combination with bed huddles at department levels [s15] and involvement of all clinics at the hospital level [s31]. A suggestion from several managers is to support these meetings with a weekly tactical capacity meeting to plan and settle disputes or misalignments [s34]. Additionally, it is important to have an internal structure for problem-solving supported by a continuous improvement culture, flow engineers, and a local operative management team [s16], as highlighted by this vice president:

*“I am a Lean management fan, and we try to have a Lean daily management approach with huddles at the unit level, the OR level, and at the ED, where teams will at least, twice a shift, assess their capacity, throughput, and staff, cascading them up to say, where are there barriers?” -* Executive Vice President, Johns Hopkins Hospital

### Optimize capacity utilization and occupancy rates

There must be a good fit between the demand a hospital is expected to serve and the available capacity and how that capacity is subsequently utilized [s1, s10, s11, s12, s14, s17, s18, s36]. Multiple managers highlight the need for recurring strategic revisions on evolving demand patterns followed by continuous adaptations on how the capacity is distributed to have correct sizes for each department [s1]. It is also important to anchor the goals of care production across the organization and base them on what each clinic and actor along the patient flow can achieve to avoid overcrowding and unnecessary bottlenecks [s36]. Furthermore, several hospitals point to the need for all healthcare managers and staff to understand the relationship between efficient flows and occupancy rates and the importance of running below the efficiency tipping point to avoid harmful congestion [s11], as described here:

*“When we improve our length of stay and our efficiency to get bed utilization down to 85 percent, we will undoubtedly have more demand. For example, we try to redirect some lower acuity patients but then we'll start saying yes to more complex patients, and we'll go back up to 90 percent again. If we then look at our occupancy rates, we can show that when we run above 88 percent, we lose efficiency. It creates a drag on the system. We lose degrees of freedom to move patients around, and it slows us down.” -* Executive Vice President, Johns Hopkins Hospital

There are great possibilities to optimize and smooth occupancy rates across the hospital by forecasting and estimating patients’ length of stay before settling on utilization plans for ORs, ICUs, and wards [s10]. To further increase predictability across operations, hospitals increasingly emphasize the need to level-load ORs with designated blocks per clinic, putting caps on the number of surgeries. This solution enables greater balance over the week and across services [s12], further explained by this patient flow director:

*“Surgeons want to operate, so avoiding OR days on Mondays or Fridays, which tend to be holidays and get cancelled, makes sense. This leads, though, to low surgical volumes on weekends and Mondays, building on Tuesdays, and potential cancellations due to high volumes on Wednesday and Thursday. This is hard on the staff and creates stress trying to get all surgeries through. We shifted to a goal that every day, there is a smoothing target by the type of surgery or units that patients will go to. When we now schedule, we proactively set targets, saying, ‘you can do five a day, and that's it. You can't go over’. That's been very effective in managing surgical flow.” -* Director, Patient Flow, Toronto General Hospital

Ensuring high utilization of the OR capacity can come from better utilization of OR days, smart mixing between short and long cases, filling the schedule from the back, and having a pool of patients for quick cancellation refills [s14]. It can also come from better long-term planning of OR schedules and surgeons prioritizing surgeries before other activities [s17]. Increasingly, pressuring demand patterns also force hospitals to better utilize the working week by introducing more flexible staffing schedules outside traditional hours to handle both present demand and sudden peaks [s18].

### Seek external solutions and policy changes

No matter how efficient internal operations become, an organization is always dependent on the wider system it belongs to for overall efficiency, and there is a need for better alignment and increased capacity across the healthcare system [s6, s8, s9, s44]. Concerning primary care, hospitals find themselves treating and caring for patients that would be better served by primary care providers and point to solutions of extended primary and urgent care presence with longer opening hours, closer hospital collaboration, and dedicated specialist-led education of general physicians [s6]. Many hospitals also find themselves squeezed between a never-ending inflow of patients and difficulties in finding aftercare providers willing to accept discharge-ready patients. The question is whose responsibility it is to care for discharge-ready patients where improved transfers may come from increased downstream bed capacity, changed legislation, or new incentive programs [s44], something seen in this interview:

*“The one who sets the agenda for when we can send a patient is the external actor. It's not us. We can kindly stand with the hat in hand and ask, ‘could you maybe take this patient?’ where the answer is, ‘no, we cannot; we can on Monday’. We have regional and municipal healthcare with too many principals, different politicians, and budgets, and they push costs on each other. It's a huge concern when it's the same patient flow, and there is a lack of common goals between these actors with regards to patient flow.” -* Medical Director, Karolinska Universitetssjukhuset

This all boils down to a need for more patient-centric care and alignment of all care providers across the healthcare system with clear task descriptions, common patient goals, and policymakers focused on transforming the system [s8]. Most hospitals also acknowledge the chronic staffing shortages in the healthcare sector and emphasize the need to increase human resources across all actors. The scapegoat for much flow inefficiency is simply insufficient staffing [s9], as explained below by one medical director:

*“Another real barrier that we are facing in many places is staffing shortages. In many cases, we've designed the system with the right amount of capacity to support the care, but we often cannot staff to our plan, and we struggle to anticipate demand.” -* Chief Improvement Officer, Cleveland Clinic

## Discussion

There is a great need for improved hospital productivity to meet the challenges of future healthcare demand, and previous research shows that more focus on patient flow can help increase hospital productivity [12, 20, 21, 26]. The system-wide perspective is increasingly emphasized as patients move between multiple professionals, clinics, and administrative units along their trajectory of care [20, 22, 29, 33, 34]. We present 50 solutions, taking a system-wide perspective on what hospitals can do to enable swifter patient flows across their organizations. Our findings show that multiple professional, cultural, managerial, technical, and political aspects must be addressed and that a holistic strategy covering patients’ whole trajectory of care is needed. The presented categories of solutions can be found in previous research concerning parts of the hospital patient flow, as needed developments, or as implemented interventions. The need for “*better organizational alignment”* is highlighted by several studies [29, 34, 36, 47, 48] to make the organization process-oriented [34] and better integrated with clear organizational goals [36]. Having *“better coordination and transfer structures”* has been identified [12, 20, 22, 31, 34], highlighting the need to have patient flow managers with strong mandates [20] and central patient and transfer coordinators [22]. *“Increased physical capacity capabilities”* [16, 19, 49], like increased investments in ancillary services [16] and the expansion of home care services [49], are important. Several studies also confirm the need for *“more standards, checklists and routines*” [20, 22, 25, 32, 33, 50] to enable more efficient capacity utilization [20] and to reduce lead times and improve medical outcomes [50]. The need for *“more digital and analytical tools*” has been found [12, 20, 21, 26] to give support to the scheduling, diagnosing, and coordination of care [21] and enable real-time data visibility [26]. Other researchers have uncovered the need for *“better management of operations*” [9, 26, 32, 50], including centralization around a patient flow management centre [26] and a stronger focus on continuous improvements [32]. Moreover, several researchers confirm the *“need for capacity optimization and occupancy rate balancing”* [7, 19, 20, 51], like smoothing the surgical schedule [19] and better predictions and avoidance of disaster-level overcrowding [7]. Lastly, previous research emphasizes the need to *“seek external solutions and policy changes”* [4, 22, 29, 34] to create more integrated healthcare systems [29] and make policymakers and politicians understand the arising staffing crisis [4].

Despite the complexity of being large academic hospitals, these hospitals succeed in implementing several of the highlighted solutions. Previous research points to top management support, one of the most important factors in successfully implementing change [22, 52, 53], as one likely explanation. We cannot, from our study, establish this direct link, but it is worth noticing that these interviews only involved senior managers. Consequently, having a top manager working with flow-related questions, and holding a holistic view of the hospital-wide patient process, likely provides important support to commissioners when improving the flow of patients. These hospitals also strategically plan their activities and improvement projects from a hospital-wide perspective, something previous research indicates is often missing [32, 33, 47]. Moreover, these hospitals are considered leading because of excellent medical performance and patient satisfaction [39], which previous research has found to be supported by swift patient flows and short lead times [21, 26].

There is an ongoing debate within healthcare services on what decision-makers and healthcare managers should do to improve the financial situation as costs continue to rise without an equivalent gain in productivity [3, 8, 9]. The question looming is whether productivity improvements can be reached with or without increasing available resources [6, 11]. This study gives good insights into the thoughts of senior managers at the world’s leading hospitals concerning the best path ahead. Multiple hospital managers consider their patient flows to be constrained by an insufficiency of beds and staffing resources. Simultaneously they highlight a myriad of projects and solutions on how to improve the processes without increasing expenditures and how to best use already available resources. Together, these hospitals consider the path forward to be both work-method-related and resource-related, saying that much can be achieved without increasing costs. Increasing available resources to meet rising demand has been tried on multiple occasions over the last decade, many times with consequences of rising costs and rarely equal gain in productivity [9, 11, 12, 19]. One recent study also projects staffing deficiencies to rise notably over the coming decade [4], further emphasizing the need to either increase available capacity or use available resources more wisely. Hence, if increased financial support might be hard to agree upon with policymakers and politicians, our study gives multiple alternative solutions to help hospitals confront the challenges of increasing demand.

Another problem lies in a seemingly unsustainable logic prevailing in healthcare of utilizing too much available capacity. This study reveals that whenever capacity is extended, additional bed and staffing resources are quickly used, reverting capacity utilization to previous levels. Managers interviewed in this study derive this phenomenon from an infinite demand and an unsustainable logic of over-utilizing available capacity. This leads to hospital-wide overcrowding, burned-out healthcare staff, and slow patient flow, and it ultimately reduces medical quality and patient satisfaction. The high capacity utilization creates “a drag on the system,” as one manager expressed it. Even though it might seem as though resources are used optimally, seen from a resource utilization perspective, the number of patients treated by the hospital is decreasing as the throughput of patients slows down. Previous research has traditionally advocated for 85% as the optimal operating occupancy level for hospitals, stating that occupancy rates above 90% slows down the patient flow across the organization [28, 54, 55]. However, Bain et al. [56] point out that the traditionally suggested 85% occupancy level target is not an optimal, one-size-fits-all measure. Some hospitals may reach their “choke-points” at both higher and lower levels. Even so, staying at high occupancy rates, above 90%, generally has a direct negative impact on hospitals’ ability to provide safe and timely services for patients [57]. However, pressing down the occupancy rates to more sustainable levels is frequently a difficult act, as the demand for healthcare services is increasing faster than the available capacity [1, 2]. Therefore, it is difficult, and many times impossible, for hospitals to say no to patients in need of care. However, as indicated here, admitting more patients might result in fewer patients being treated and, ultimately, reduced public health. Hence, adding more resources without improving the work methods and the logic of capacity utilization seems to only make hospitals repeatedly end up in the same situation. Focusing only on method improvements might be unreasonable, as there is little “free” capacity to spare for ambitious improvement projects. Consequently, this points to a strategy of building capacity to provide sufficient margins to the organization and then use that capacity to improve work methods and change the capacity utilization logic. This may improve the flow of patients, provide better and safer care for more people and enable a more sustainable work environment for healthcare professionals.

### An improvement framework

**Fig. 2:**
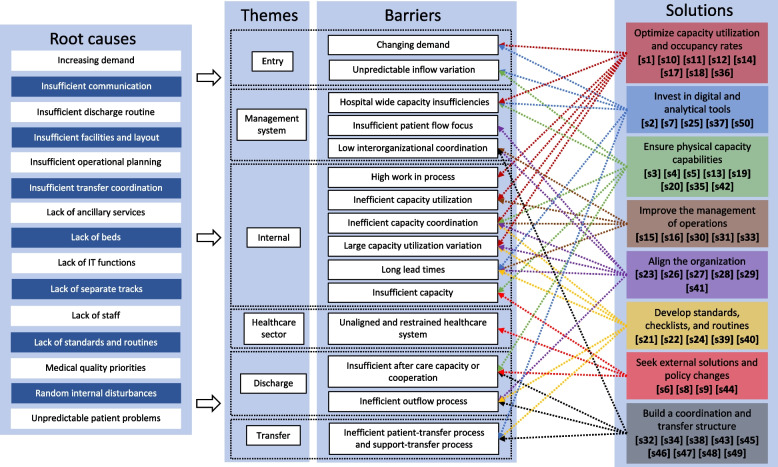
The patient flow improvement framework

It is difficult to make the patient process more efficient, and it is hard to identify the path forward in the complex environment of the modern hospital organization. To address this, we have developed a patient flow improvement framework of themes, barriers, root causes, and solutions; see Fig. 2.

The framework highlights several themes to direct readers to how and where patient flow barriers may appear across the hospital, supported by Fig. 1. The solutions are presented in Table 3 and supported by  Additional file 1: Appendix A, where Åhlin et al. [40] explain the causal relationships between barriers and root causes. Hospital managers and commissioners may take several different approaches when using this framework. The framework can be used by identifying a certain root cause or barrier and then looking for appropriate solutions to implement. Another path might be to start with the desired solution and explore how and where that solution will impact the organization. A third approach could be to select a certain part of the patient flow, represented by themes, and see the associated problems and solutions. Hence, this framework serves as guidance for commissioners designing improvement strategies. Other frameworks or models to understand barriers and enablers to efficient hospital-wide patient flows can be found focusing on performance indicators [12], paradoxes of patient flow [29], Lean healthcare applications [33], and patient flow improvement strategies [22]. We believe our framework evolving from this interview study and the previous systematic literature review by Åhlin et al. [40] complement their work, and these frameworks can be used together to improve the patient flow across hospitals.

### A hospital patient flow improvement plan

**Fig. 3:**
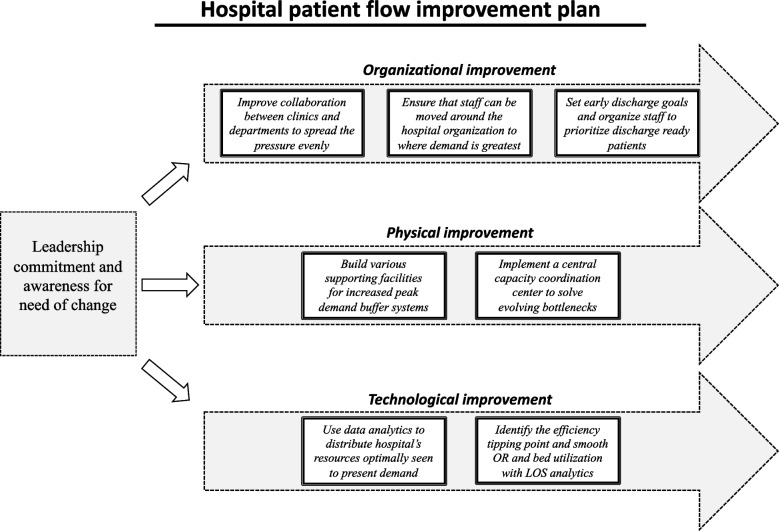
The hospital patient flow improvement plan

For hospital managers exploring the extensive list of solutions presented in this study, many of the solutions may seem too complex to implement, requiring much external cooperation and coordination. It is then closer at hand to start with solutions that only require improvement commitment within the local hospital organization. We, therefore, suggest a hospital patient flow improvement plan, highlighting what hospitals can do today without external support or collaboration; see Fig. 3 below. This improvement plan consists of three parallel improvement procedures of organizational, physical, and technological nature: *Organizational Improvement 1:*An improved collaboration between clinics and departments is necessary to spread the pressure evenly across the organization to avoid overcrowding and burned-out staff; *Organizational Improvement 2:* Staffing pools or interim personnel units are needed to ensure that staff can be moved around the hospital organization to where demand is greatest; *Organizational Improvement 3:* To better balance available bed capacity with the arrival of admitted patients, clinics must become better at setting early discharge goals and organising staff to prioritize discharge-ready patients; *Physical Improvement 1:* It is necessary to have an efficient central capacity coordinator, like a command centre, that oversees the capacity situation in real time and can act with a strong mandate to solve evolving bottlenecks; *Physical Improvement 2:* It is important to have various facilities that can handle sudden surges in demand, like patient hotels, discharge lounges, short-stay units, and temporary extra wards to enable buffer systems; *Technological Improvement 1:* There is a need to understand the efficiency tipping point of the hospital and to work with OR planning based on the downstream bed (ICU/ward) availability, block schedules, and surgical smoothing; *Technological Improvement 2:* It is important to assure, through data analytics and strategic capacity revisions, that the hospital’s resources are distributed optimally to present demand. These perspectives demonstrate a need for hospitals to build organizations that proactively and reactively optimize capacity use around patient flows to deliver healthcare services for as many as possible and to spread the burden on healthcare professionals evenly across the organization.

When initiating an improvement plan, research on change management highlights the need for leadership commitment and an awareness of a need for change throughout the organization to enable sufficient support for the process [53, 58]. Glouberman and Mintzberg [31] and Radnor et al. [32] also explain the need for healthcare professionals to move away from the prevailing silo mindset and to take a more holistic responsibility for the purpose and outcome of the whole hospital system they belong to. We believe the processes described in this improvement plan may help increase healthcare professionals’ awareness of the impact of their choices on patient flow across the hospital.

### Contributions and limitations

This article gives insights on how to improve patient flows across hospital organizations. It provides concrete guidance to healthcare managers, commissioners, and decision-makers on what solutions to focus on and the barriers and root causes they are helping to overcome to provide the right care to as many patients as possible. Using a wider lens, our study provides new perspectives on the contextual and causal complexities of patient flows across hospital organizations. We encourage practitioners to approach patient flows from a hospital-wide perspective and encourage researchers to explore more aspects of the hospital-wide challenges and possibilities to improve the flow of patients across hospitals. The findings of this study call for research on how solutions for improved patient flow efficiency are best implemented and how hospitals best prioritize their hospital-wide patient flow improvement strategy. Additionally, we suggest more research on the perceptions of other professional groups across the hospital on the organizational development toward more efficient hospital-wide patient flows.

This study comes with some limitations. A research method of qualitative semi-structured interviews was used, with a substantial pilot study conducted beforehand. Even so, the interviews were conducted by a single researcher, creating a risk of subjective bias and perspectives when posing questions and guiding interviewees. Another limitation is associated with the online format, as body gestures and facial expressions are harder to capture in a non-physical setting, limiting the possibility of fully acquiring the answers and views of the participants. Furthermore, even though the thematization was conducted by three researchers in which everyone independently read through the transcribed interviews, our common background as researchers within the same field may limit our frame of reference and the width of possible interpretations. Additionally, we have large academic hospitals in this study, and their views on the most appropriate path to achieve efficient patient flows may not be useful for all types of hospitals. As such, for a more complete view, this study would need to be complemented by research on hospitals with other characteristics, like secondary care providers. Moreover, in this study, only managers were interviewed, highlighting the need to explore the hospital-wide patient flow from the perspectives of other professional groups, such as physicians or nurses working directly with patients.

## Conclusion

To optimize the patient flow across the whole healthcare organization, hospitals must employ a wide array of solutions. Multiple professional, cultural, managerial, technical, and political aspects must be addressed, and a holistic strategy that covers patients’ whole trajectory of care is needed. Hospitals must proactively and reactively optimize capacity use around their patient flows to ensure higher productivity and a better working environment. This study concludes that the efficiency of internal hospital-wide patient flows largely depends on collaboration and cooperation with external actors, highlighting the need to improve the flow of patients along the whole healthcare value chain. Even so, much can be done internally by the single hospital through a focus on relevant organizational, physical, and technological issues. This study also shows that even though the scapegoat for flow inefficiency at hospitals may be insufficient staffing, hospitals can do many things to improve the throughput of patients without increasing expenditures. Lastly, hospitals across both Europe and the US share, to a large degree, the same view of the path forward, indicating that the solutions on how to improve hospital-wide patient flows apply to many hospitals and healthcare systems.

## Supplementary Information

**Additional file 1: Appendix A.** The Interview Guide.

**Additional file 2: Appendix B.** Open coding of interviews.

**Additional file 3: Appendix C**. The number of hospitals that acknowledges the need to work with a particular solution is categorized from the highest to the lowest number together with the name of the solution and associated theme.

## Data Availability

All datasets generated or analyzed during this study are included in the main manuscript (and its supplementary information files).
