# Supplementary material for: Solutions for improved hospital-wide patient flows – a qualitative interview study of leading healthcare providers
Source: BMC Health Serv Res. 2023 Jan 7;23:17. doi: 10.1186/s12913-022-09015-w (PMC9825009; doi:10.1186/s12913-022-09015-w)
Supplement: Supplementary file 1 — Additional file 1: Appendix A. The Interview Guide. [file 12913_2022_9015_MOESM1_ESM.pdf]

## Appendix A: The Interview Guide

1. What do you do to improve the coordination among the different clinics of the hospital, as a means of improving the patient flow?

2. What do you do to improve the flow out from the hospital, and to enable an efficient discharge process?

3. What do you do to improve the flow into the hospital considering both the acute care as well as the planned care.

4. What do you do to improve the transfer of patients between different clinics at the hospital, as well as between patient responsible clinics and ancillary services?

5. Zooming in on the single clinics, how do you work with meeting their capacity requirements?

6. What do you do to make sure that the available capacity is utilized efficiently, is coordinated in the best possible way, and that the utilization of the available capacity is not varying disproportionally?

7. What do you do to decrease overcrowding at your hospital and how do you prevent having too many patients in treatment at the same time?

8. Concerning the patient flow within the single clinics, what do you do to decrease their lead-times?
