# Supplementary material for: Solutions for improved hospital-wide patient flows – a qualitative interview study of leading healthcare providers
Source: BMC Health Serv Res. 2023 Jan 7;23:17. doi: 10.1186/s12913-022-09015-w (PMC9825009; doi:10.1186/s12913-022-09015-w)
Supplement: Supplementary file 2 — Additional file 2: Appendix B. Open coding of interviews. [file 12913_2022_9015_MOESM2_ESM.pdf]

Appendix B: Open coding of interviews

Themes	Solution Categories	Open Interview Codes
Discharge	Enable discharge predictability with more home care solutions and own downstream facilities	A good home care solution is to have the hospital responsible 3 days, therafter GPs
		A whole separate team handles the home care patients
		Build after care capacity to support hospital
		Build out home care possibility as it gives many good options to the hospital
		Build out home care to avoid bottlenecks with rehab or nursing facilities
		Have a good home care organization of nurses and physicians
		Home care can strengthen the care and the relationships
		Home care solutions - 10 nurses and doctors work in the team to see patients daily
		Home care solutions - hospital responsible for a week
		Home care solutions can finish IV antibiotics at home and require low nursing needs
		Hospital staff visit discharged patients in their home to release bed capacity
		Hospitals can cover the entire continuity of care
		Improve virtual care through in-home monitoring
		Inpatient team follows patients at home for continuity
		Invest in home care and cooperate between hospital, GPs and community
		Invest in own downstream facilities
		It is good to have much home care capacity
		Release bed capacity by supporting the patient back home
		Set up a camera to virtually see and monitor the patients in their homes
		Take more responsibility for after services
		The capacity of the hospital can increase if home care solutions are improved
		Use a designed home care program to facilitate change
		Use home care solutions to free up space and bypass after care services
		Work with home care to ease the burden on the hospital
	Have dedicated discharge coordinators or coordinating teams	Case managers and social workers coordinate the outflow
		Case managers are right in the middle of the coordination of the discharges
		Case managers personnel help to plan for discharge
		Financial officers to support flow to ensure insurance companies are connected
		For discharge efficiency make physicians work as a part of the team
		Have multidisciplinary rounds every day with proactive case managers
		Operative direction coordinates discharge with after care
		Outflow care management team handles discharges
		Patient care coordinator responsible for discharges
		Smoother discharge processes by daily rounding structure with care coordinators
		Social assistant who coordinates after care
		Social worker ansd care coordinator responsible for patient's needs
		Social worker manages discharges to the home, case manager coordinates facility discharge
		Use coordination consultants in the discharge process to facilitate transfer
		Ward physician responsible for discharge flow
	Increase prioritization of discharge ready patients when planning procedures and activities	Base MRI-radiology schedule on discharge readiness to expediate flow
		Discharge in early morning, teach in the afternoon
		Identify limiting step to discharge
		Milestone target perspective for each patient
		Physicians must prioritize discharges
		Prioritize discharge procedures
		Prioritize flow before severeness more often
		See if tests can be prioritized for discharge ready patients
		Smooth discharges by improving week-end procedures
		Strong daily focus on discharge ready identification
		Try to release discharge ready patients by prioritizing their last steps
		With real time data prioritize discharge ready patients at ancillare services
	Prioritize activities and organize staff to ensure early and efficient daily discharges	Deliver first week of medicine at inpatient pharmacy to expediate discharge
		Discharge before noon by changing morning routines
		Discharge before noon using KPIs
		Discharge directly from the recovery room
		Discharge lounge to free up beds for cleaning
		Discharge teams to meet every day to discuss and expediate discharge
		Encourage and incentivize morning discharges
		Give time and support to physicians to expediate discharge
		Have daily discharge synchronization
		Medication at discharge to avoid readmission or medication outside to improve flow
		The EHR automatically imports info to give a codified discharge summary
		Use a common medicin card for better discharge
		Use a discharge lounge at the ground floor for discharge ready patients
	Provide follow up appointments at discharge to ensure accountability and continuity	Discharge team has access to outpatient schedule to schedule follow up appointment
		Follow-up appointment must be booked at discharge to avoid readmission
		Have standardized phonecalls with nurse practitioners after discharge to ensure medical quality
		Make sure there is a primary care appointment booked at discharge
		Provide follow-up appointments at discharge
		Provide tele consultations with patients in after care to avoid readmissions
		Receive follow-up information back home
		Social assistance will coordinate outpatient follow up at discharge
		Support discharges by offering a follow up video visits

Themes	Solution Categories	Open Interview Codes
Discharge	Request and work towards increased responsibility from after care services	Community has responsibility to find after care solution<br>Downstream actor accept patients when hospital needs it<br>Downstream actor must have peak-surge capacity<br>Good to have federal coordination of after care<br>Incentivize downstream transfer by giving fines to after care if long transfer lead times<br>Legislation to incentivize discharge<br>More capacity is needed in after care for complex patients that need facility discharge<br>Need for more downstream capacity<br>There is a shortage of available capacity at the facilities outside the hospital
	Set early discharge goals and work towards them along the patients' whole hospital journey	Create pre-visibility of estimated day of discharge<br>Early after care involvement - week ahead probability of patient discharge<br>EDD follow up to provide early discharge information to after care<br>Estimate day of discharge and work towards that metric<br>Every department at hospital estimates day of discharge of their patients<br>Have entire team focusing on reaching EDD<br>Improving EDD by statistics feedback<br>Organize care team to meet discharge goal<br>Plan for a specific day of discarge and give feedback on the results<br>Start planning discharge early, based on estimated day of discharge<br>Try to get a picture of when patients will be discharged and follow up on that and measure<br>Use better tools and technologies to standardize the internal discharge process<br>Use data analytics to help physicians becoming better at estimating day of discharge<br>Visualize day of discharge in EHR so everyone sees and understands the process
	Share objectives, information and real time capacity data with after care services	Align objectives with after care through regular meetings on needs and quality<br>Annual convention with after care on patient flow<br>Build good relationships with after care<br>Coordinate closely with a selected group of rehab centers, nursing homes and nursing facilites<br>Have a clear transfer of accountability to after care<br>Have good electronic communication with after care<br>It is necessary to have good relationships with after care services<br>Mutual agreement with after care on patient care chains<br>Occupancy data sharing between hospital and after care<br>Partnership with after care services<br>Share medical records with after care services<br>The most important thing is to have a good relationship with the players who take over the patient<br>Try to understand each other - after care and hospital<br>Use a discharge summary for chain of accountability<br>Work closely with managers of rehabilitation to find good solutions for patients
	Use mutual staffing collaboration between the hospital and after care services	After care has offices and staff at the hospital<br>Build closer relationships by staffing nursing facilites for coordination<br>Have nurse practitioners who frequently visit nursing facilities<br>Nurses from public after care service are present at the hospital for coordination<br>Send nurses and doctors with patient to give releif to after care<br>Staff nursing facilites for better coordination<br>Use hospital staff at nursing facilities<br>Use outgoing teams to after care<br>Work with nurse to nurse hand off with after care<br>Work with physician to physician handoff with after care

Themes	Solution Categories	Open Interview Codes
Healthcare sector	Create healthcare system alignment with clear goals and objectives for each healthcare actor	A more jointly coordinated health system is needed<br>Common patient goals between all healthcare actors<br>Create a patient centric care across all healthcare actors<br>Create a systemness to the healthcare system<br>Crisp task descriptions for primary care, hospital and after care<br>Policy makers must understand the costs coming with having peak time capacity<br>Politicians must support the investment of more ICU beds to avoid overcrowding and bottlenecks
	Increase the staffing resources across the whole healthcare system	After care services are understaffed, hospitals are understaffed, primary care is understaffed<br>Find ways to deal with staffing shortages<br>Healthcare needs more staffing resources<br>If we do not have enough staff it does not matter if we improve our processes<br>More specialist nurses are needed in many different areas<br>More staffing resources are needed across the whole system<br>Our staffing resources must increase for us to speed up the flow of patients<br>The bottlenecks are very often associated to staffing shortages<br>The hospital as well as after care services must increase the staffing<br>To improve the flow we must employ more nurses<br>We must both find ways to work with less staff but people are simultaneously very important<br>We must educate and employ many more nurses in the whole healthcare sector<br>We must find ways to deal with the staffing shortages even if it is hard<br>We suffer from chronic staffing shortages and it really has an effect on the patient flow

Themes	Solution Categories	Open Interview Codes
Entry	Capability to reroute less severe ED patients to other types of care	Admission discussed with flow officer to look for alternative routes<br>Analyse demand to avoid admissions by rerouting patients to more appropriate settings<br>Close communication with outpatient clinics to avoid physicians sending patients to the ED<br>Have a system to support planned acute surgeries<br>Invest in front end tools and use pathways to shape demand and avoid ED admittance<br>Prevent less severe acute patients from coming by changing incentives<br>Redirect less severe ED patients to outpatient locations and inform primary care<br>Transfer less severe ED patients to outpatient appointment
	Cooperate with other hospitals to ensure bed capacity and to seek appropriate level of care	Coordinate occupancy levels between hospitals in the region to balance load<br>Give coordinating emergency number visibility on hospital occupancy<br>Improve intra-hospital coordination by bringing sompler cases to other hospitals<br>Improve intra-hospital coordination by enabling right level of care<br>Improve intra-hospital coordination by relocating patients to the right hospital with the right level of care<br>Let external actor coordinate and balance the flow of patients to the different hospitals<br>Outsource or hand over simpler care to other hospitals<br>Plan real time for an optimal capacity between nearby hospitals to smooth load<br>Push secondary care to community hospitals<br>The most advanced hospitals cannot be everything to everyone<br>Use alert signs at hospital website to direct ambulances to hospitals with most capacity<br>Use intra-hospital coordination of the bed capacity
	Reach, inform and treat patients before they seek acute hospital care	Communicating hospital capacity using defined community distribution lists<br>Have a virtual ED before real ED<br>Have hospital off hours 24-7 hotline<br>Make outpatient teams communicate to patients, expectations on ED waiting time<br>Mobile teams meets patients in their homes<br>Prehospital call line secures acute patients are arriving to a hospital with capacity<br>Prehospital has mobile teams who reach and treat patients to avoid hospital care<br>Provide a tele-medicine hospital to support patients at home<br>Send preventive information to the public when capacity is restrained<br>Use an online express care as an initial triage<br>With info on patient google searches, hospitals can reach out to patients at an early stage in their sickness
	Require increased primary care responsibility and support with more knowledge exchange and coordination	Cooperate and invite primary care doctors to teach them to take more complex patients<br>Coordinate strategically with urgent care providers to avoid ED admissions<br>Educate the GPs in areas where they send too many patients<br>From a societal perspective more care should be given by primary care physicians<br>Help to make primary care physicians take more patients<br>Make GPs write better medical referrals<br>More collaboration with primary care physicians is needed<br>Primary care education and coordination<br>Primary care must have longer opening hours<br>Primary care must have longer opening hours and better availability<br>Primary care need to take more patients<br>Teach the physicians in primary care to treat patients with more complex problems<br>Urgent care plays an important role in offloading the emergency department
	Strategic planning: make recurring strategic revisions on fit between demand and capacity	Build annual makro model on demand and available capacity<br>Central management are inolved to strategically look at capacity gaps<br>Central management make quarterly reviews on unused OR capacity<br>Central management strategically looks at capacity gaps<br>Closely monitor demand and capacity to identify misfits for certain disciplines<br>Forecast demand - capacity requirements four times a year<br>Make long term planning on demand and hospital capacity<br>Management takes decisions on employment of critical specialities to fit with demand<br>Reoccuring capacity use problems are solved by the top management<br>Set capacity across hospital based on annual revisions on the need of the community<br>The central operational group studies utilization of outpatient clinics on capacity and backlog<br>The hospital managment must handle unresolved issued between the departments<br>Unsolved problems must be taken care of by the top management<br>Use demand forecast models with LoS, market share, to establish occupancy rates<br>Use predictive analytics to right size departments
	Strategic planning: use predictive analytics to forecast demand patterns and capacity needs	"Seven day hospital"- Use data to identify capacity need all seven days of the week<br>Build predictive views on scenarios for certain plans and demand patterns<br>Create situational awareness by using predictive data to estimate bottlenecks<br>Develop better outpatient frontend analytics<br>Develop predictive analytics to anticipate need of capacity margin<br>Develop predictive analytics to describe likely patient flow and requirements<br>Develop predictive analytics to forecast bottlenecks and overcrowding<br>Develop predictive analytics to have proactive trigger points<br>Develop predictive analytics to optimize staffing<br>Gather patient flow data to understand patterns<br>More predictive analytics could be used for the patient flow through the outpatient clinic<br>Provide forecasted risks concerning planned admissions and discharges<br>Use algorithms to predict capacity need<br>Use better tools to anticipate future demand<br>Work proactively to anticipate problems or bottlenecks
	Use IT-tools and data-analysis for standardized admissions, early assessments and reduced no-shows	Better time planning can reduce wait for pre-assessments<br>Digital pre-assessments by patients<br>Have a preoperative strategy assessing patient on video or by phone<br>Have a single phone number for each clinic to reduce patient confusion<br>Make patients accountable for outpatient no-shows<br>Reduce variability in ED admittance by visualizing practice variation among clinicians<br>Use robots to read and sort referrals<br>Use standardized referral pathway for efficient handoffs and communication

Themes	Solution Categories	Open Interview Codes
Internal	Allocate dedicated capacity for both acute and elective patient flows	Acute and elective surgery must be separated to not cancel surgery<br>Caps on the OR schedule to secure capacity for acute patients<br>Have acute and planned care on separate floors and within separate surgery divisions<br>Operate acute cases immediately to better use OR and push flow<br>Separate acute and planned surgeries<br>When deciding capacity for elective OR cases, have a sufficient margin for acute cases
	Ensure a high OR-utilization with smart case mixes, all day utilization and quick cancellation refill	Begin with long cases and after short to utilize whole day<br>Count used and unused slots for surgery and appointments to improve utilization<br>Fill schedule from the back<br>For OR use, start early first case on time and optimize changeover times<br>Have a pool of patients on SMS-list to find a patient if a surgery is cancelled<br>Have capacity alerts to bring in patients on the waiting list<br>Improve the OR use after 15 to improve capacity<br>Make the OR staff understand need of flexibility around last case of the day<br>Make the staff understand the need of being flexible around the shedule to finish the cases of the day<br>Plan OR schedule strategically with both long and short cases over the day<br>Plan the OR schedule beginning with the long cases and contine with short later in the day
	Have a structured organization for daily problem solving and capacity optimization	Employ flow engineers to visualize and break bottlenecks<br>Have an operating management team to improve time use, changeover and processes<br>Hospital culture must shift to continuous improvement culture<br>It is important to have a problem solving meeting structure<br>Make change or improvement seen as good and necessary<br>Once a month there is a meeting about the present operations to solve bottlenecks<br>Tactical planning meets once a week to look ahead<br>Team for bottleneck identification<br>Use flow engineers to identify patients choke points
	Improve outpatient processes by implementing standards on schedules and appointments	Clear outpatient clinic standards on clinic time, start-stop, amount of appointments<br>Have outpatient clinical slot standardization<br>Same speciality clinics should have standardized set ups<br>Standardized physician times per patient in outpatient clinics<br>The outpatient world must become more organized like the inpatient side<br>Use room or template utilization to improve outpatient capacity efficiency
	Improve prioritization schemes and develop standards on procedures, roles and staff-bed ratios	Create clear prioritization lists among work tasks<br>Create flexibility across the hospital by working more with standardization<br>Create standardization with electronic instructions system<br>Greater utilization, lower variability, less staffing flexibility<br>Have a common and standardized coordination of the surgery process<br>Have clear guidelines for prioritizations on when to cancel surgeries<br>Have clear roles and responsibilities<br>Management must set clear prioritization schemes for surgeries of same type when capacity is filled<br>Practice to the top of your license<br>Standardize routes of communication<br>Standardize treatment and reduce artist culture<br>Surgeon puts patient on OR waiting list, head of department decides priority, nurse coordinator schedules the patient<br>Translate volume into resource equivalents to evaluate capacity<br>Use clear nursing rations per bed, speciality or type of patient<br>Use clear targets for nursing and bed ratios
	Invest in ancillary service capabilites to minimize bottleneck risks in indirect patient	Build lab capacity to deliver lab results at 10 a.m. every day<br>Expand sterile central to improve equipment predictability<br>Invest in diagnostics capacity<br>Need of more radiology capacity
	Make all employees understand the importance of having a patient flow focus	Change culture from personal preferences to flow prioritization<br>Change staffs individual preferences to a flow incentive<br>Individuals must understand the patientflow<br>Make doctors aware of the consequences of their decision on the flow<br>Making staff responsible for the throughput of patients instead of the single activities<br>Show everyone that uninformed capacity decisions can lead to flow inefficiencies<br>Staff must understand implications across processes
	Operational planning: have daily capacity meetings within the department or clinic	Daily meeting on capacity, anticipated ins and outs<br>Daily organizational bed huddles with patient care managers and charge nurses<br>Daily steering with use of input and discharge boards<br>Organize staffing capacity in morning and afternoon to synchronize with demand<br>Reoccuring capacity meetings during the day to solve bottlenecks
	Optimize and smooth occupancy rate levels by admitting patients based on length of stay and ICU risk	Base bed occupation on length of stay<br>Base capacity on optimal occupancy rates and estimated demand<br>Base OR case mix on available beds and anticipated LoS<br>Base planned care on anticipated occupancy census level and implications on ED boarding<br>Forecast how beds open up based on estimated LoS<br>Forecast patient mix and LoS to identify necessary beds and staff<br>Improve flow by both building OR schedule on LoS and have fluid caps on ward beds<br>Make the beds evenly full over the week by building OR schedule based on estimated LoS<br>Optimize OR schedule with a mix of specialities to level load occupancy and severeness after surgery<br>Optimize system around capacity usage<br>Optimize the system around the OR use to minimize variation and increase efficiency<br>Plan elective care based on LoS to smooth capacity<br>Set up OR schedule based on likelihood of ICU treatment<br>Show what type and how many patients clinics will have so they understand difficulties of the ICU<br>Use daily risk estimation to decide operations flexibility<br>Use predictive analytics to estimate LoS for optimal scheduling<br>Use predictive analytics to estimate LoS for optimal scheduling

Themes	Solution Categories	Open Interview Codes
Internal	Schedule staff and all clinical activities based on an optimal utilization of the OR-schedule	Align flow by adapting OR schedule, clinic schedule and research schedule<br>Build surgeon schedule around OR-efficiency<br>Decrease OR variation by making surgeons priroitizing the OR-schedule first<br>Find balance between OR efficiency and surgeon efficiency<br>Identify demand patterns for scheduling<br>Make surgeon plan research, teaching and appointments after whats best for the OR-schedule<br>Plan the staffing schedule well ahead<br>Surgeons must prioritize surgeries before their research
	Give clinics trust and improvement autonomy but follow central process metrics and external benchmark	Benchmark for internal production efficiency<br>Clinics are responsible for mapping out and organizing their beds<br>Clinics independantly set goals and use metrics to reach them<br>Compare standards and ratios to national benchmarks<br>Each outpatient clinic manages the flow within their clinic<br>Efficiency is driven by measuring length of stay metric<br>Identify capacity need by measuring time to finish activities<br>Identify capacity requirements based on internal capacity revisions and external benchmarking<br>Improve lead times with PREM<br>Look at beds available versus beds filled and amount of appointment slots filled<br>Look at internal metrics to ensure internal efficiency<br>Look at surgeon operating time to improve flow<br>The key metric on efficienecy is length of stay (LOS)<br>Use detailed follow-ups on metrics for efficiency<br>Use internal process metrics for efficiency
	Understand the tipping point of hospitals' capacity utilization and ensure sufficient capacity buffers	Define optimal occupancy rates and work towards them<br>Know your tipping point on occupancy and flow<br>Make sure to run below the critical capacity utilization level<br>Measure the use of the ORs closely to be between 80-85%<br>Plan surgical schedule with high margin to avoid peaks<br>Run below the occupancy rate tipping point where efficiency decreases<br>Run below the occupancy rate tipping point where efficiency decreases<br>The limitless demand logic must stop<br>There must be limits to how overcrowded a hospital can become<br>Understand where you have your capacity tipping point
	Use external facilities or patient hotels to release hospital bed capacity	Have a patient hotel to release capacity<br>have transplant patients living in external facility during treatment<br>Use a patient hotel when the patient is not sick enough to need an inpatient bed<br>Use patient hotel to avoid admitting patients to the hospital
	Use more digital tools and new treatment methods to reduce lead times	Create more capacity for ambulatory - day care services<br>Create more possibilities for the day hospital and care in the outpatient departments<br>Digitalization to make care more transparent to patients and staff<br>Have X-rays ordered digitally<br>Invest in new technology to enable less treatment<br>Move treatments from inpatient to outpatient if possible<br>Reduce internal variation locally with data support from central management<br>Transform care into more ambulatory care and build that capacity<br>Use ERAs (enhanced recovery after surgery) to make patients ambulate faster<br>Use shared patient dressing rooms to save time before treatment
	Use an OR block schedule per clinic and plan cases based on downstream bed availability	Block schedule for each department and if not used then open up for others<br>Build OR model with expected vacation, sick leave and available beds.<br>Check periodicity to ER volumes and then schedule elective cases to counteract or offset that<br>Create pre-visibility to plan OR schedule based on typology of patients and availability of beds<br>Define flow for different patient groups to optimize whats controllable and balance against the uncontrollable<br>Have a few surgeries per day per department to smooth<br>If beds are filled up then the flow team can prioritize to use OR for ambulatory patients<br>Level load capacity over whole week<br>Maximize use of the OR to level load across week<br>Need for operative scheduling across the services for level loading<br>Postpone an elective case until bed availability is guaranteed<br>Set caps on amount of surgeries according to available beds afterwards to avoid PACU boarding<br>Smooth the OR schedule and postpone cases to next week if OR becomes to busy<br>The scheduled surgeries must better match bed availability<br>Use an OR smoothing target every day for each clinic
	Utilize as much of the week as possible and staff day and week according to real demand patterns	Address cultural issues and try to utilize all days capacity<br>Allocate ED staffing resources from 12-16 to 17-22 instead<br>Base length of operating week on demand and impact<br>Create a new compensation system so staff can work outside traditional office hours<br>Have flexible staffing to adapt to peaks and valleys<br>Have more staffing in evenings and on weekends<br>Have physicians on call for decisions<br>have surgeries six days a week to level capacity<br>Improve the use of capacity all through the week. Fridays afternoon, evenings, weekends<br>Increase weekend flow by increasing weekend inpatient procedures<br>Plan clinic for variable occupancy<br>See if physicians can do surgeries in the evenings

Themes	Solution Categories	Open Interview Codes
Management System	Align objectives, metrics and patient data systems (EHR & CRM) across the organization	Coordinate systems more through an aligned EHR
		Develop an operating model (how to work with flow)
		Electronic patient journal connects forecasts on patients, where they will end up
		Have a CRM-system to give same view on patient journey and needs across hospital
		Have all metrics of the hospital on one board
		Have doctors employed by the hospital
		Have only one board of directors
		Have physicians under same organization
		Investing in new EHR system to better align organization
		Systemness by having only one hospital board
	Build up flexible hospital-wide capacity to handle peaks or capacity unbalances	By historical trends try to staff according to when there is demand
		Have a floating pool of nurses that can work in many different areas
		Have a special nursing reserve unit with nurses that can be moved around
		Have staffing resurces for demand peaks
		Having a pool of flexible staff to support where needed
		Use a central nursing staffing office to float people between services
		Use a nursing block to move nurses between clinics
		Use a surgical short stay unit (SSU)
		Use interim personnel to handle demand peaks
		Use more coordinators in evenings, weekends and holidays to have consistent efficiency
		Use multi-speciality wards for more flexible bed capacity
		Use non-traditional post anaestesia care units or PACUs of the hospital is full
	Put patient flow focus on top of the agenda across the hospital, to change the culture	Avoid overcrowding to be able to look ahead and plan the operations
		Avoid overcrowding to not always have to be in the fire fighting mode
		Change culture from accepting chaos to working with standards and weekly load balancing
		Communicate carefully and walk through future state on how to reach it safely
		Create flow incentives and levers for behavior
		Create incentives around flow and not only revenue
		Develop mindset to look across the hospital for capacity
		Doctors and all emplyees must be well tied to the hospital to care for the whole process
		Every employee must work for the whole hospital and the whole patient process
		Hospital must plan around patient flow and not be provider centric
		Implement more clinical pathways for better flow
		Make everyone understand that they have to think about whats before and what comes next
		Make organization understand that overcrowding counteract flow and revenue
		Shift culture to support patient flow
	Connect managers and staff across the hospital to break silo mindsets	Align management across organization
		Break system silos with a more flat structure
		Bring all care contacts together to break silo mindset
		Build management boards to coordinate between departments
		Connect management teams inpatient - outpatient
		Gather staff from different disciplines to give them knowledge about each other
		Managers must represent the hospital before their department
		Managers need good system understanding
		Managers should focus on surrounding actors
		See the whole hospital as a unified resource of beds
		You need management committment to change
	Ensure sufficient capacity along the whole patient flow when setting objectives to avoid bottlenecks	Assure ability of each clinic and visualize capacity use with Power BI
		Coordinate clinics along patient flow capacity to avoid resource leackage
		Create capacity by higher throughput, more capacity or less admissions
		Ensure hospital wide capacity before confirming targets
		Ensure that there is available capacity all along the flow
		Have an overcrowding crisis organization for optimal bed placement
		Plan capacity with margin to avoid overcrowding
		Plan operating theatres flow by management team
		Plan the capcity to avoid overcrowding as far as possible
		Plan the system to avoid overcrowding and back up of processes
		Set indicative mission and iteratively anchor goals across organization
		Shake hand on volumes along the flow to secure throughput
		System wide capacity planning and accountability
		The PACU can be used when the hospital suffer from overcrowding
		Use emergency wards that can accomodate patients when there are great suddens demand peaks
	Operational planning: have daily capacity meetings with all clinics or departments of the hospital	Daily capacity meetings between flow team and ICUs
		Daily inflow-outflow coordination with surgery areas
		Daily meetings with departments on beds, staff and potential transfers of patients
		Daily morning huddles for patient flow based on command center data template
		Early operational capacity meeting to see acute flow and decide upon elective cancellations
		Have a daily coordination meeting to plan the capacity and get visibility
		Have a lean manangement approach with daily huddles at unit and hospital level
		Have daily operational capacity conference
		Managers at the patient flow department coordinates daily operational meeting with all services
		The ED leads the daily capacity meeting on how to place patients

Themes	Solution Categories	Open Interview Codes
Management System	Share and visualize correct data across the organization to make everyone understand flow implications	Create visibility to make staff understand that demand is not infinite and can be managed by systemness<br>Culture can change by having and making everyone believe the numbers<br>Important to create a real time view to avoid misunderstandings and whitewashing<br>Shared visibility creates transparency and breaks silos<br>Shared visibility is needed between outpatient and inpatient settings to facilitate flow<br>Visualize data, logic for change and emotional case for change to improve flow<br>Visualize the annual volume targets per month and per day along the whole chain of care<br>Visualizing for everyone that someone has empty beds helps alot
	Tactical planning: have weekly capacity coordination meetings with all clinics of the hospital	Data supported department management coordination meetings across hospital every week<br>Having OR meeting one week ahead with accurate and real data to optimize planning ability<br>Operational direction provides OR mission two weeks ahead, clinic then assess resources<br>Provide weekly understandable plans to clinics
	Use a flow command center to optimize capacity use and break hospital flow bottlenecks	Air traffic control coordinates rooms, admittances and discharges<br>Air traffic control oversees flow and acts on bottlenecks<br>Control rooms gives global picture and vizualises and acts on capacity imbalances<br>Coordinating center manages the flow and identifies bottlenecks<br>Coordinating center takes care of administration and documents<br>Coordinating room can see and act on flow bottlenecks<br>Flow department has patient deck list and fills treatment schedule when they see unused capacity<br>Flow department level loads surgeries globally<br>Flow department meets with OR daily to smooth OR cases based on anticipated bottlenecks<br>Flow department optimizes daily utility<br>Have a bed coordination center to optimize capacity and capability<br>Have a physical command center for capacity management, cleaning services and nursing staffing<br>Hospital wide operational coordination service (direction d'operation)<br>Operations command centers are important to have<br>Physicians and flow department give each other awareness of decisions<br>The flow department manages surge beds<br>Use a command center to coordinate beds, staff and bottlenecks<br>Use command center together with aligned EHRs and bed management systems<br>Use command centers like air taffic control rooms
	Use IT-tools to analyze bed capacity use and provide daily real time visibility on hospital capacity	Connect bed capacity with staffing resources real time<br>Have a dashboard with available beds and staff for each unit<br>Have good automated systems for statistics is important<br>Have operational metrics on the daily boards<br>Have screens with real time discharges - admittances<br>Provide better insight to bed capacity<br>Provide real-time information to operating leadership<br>Robust real time information about current situation<br>Send out a situational report twice a day<br>Use data analytics to see evaluate capacity in terms of use of beds and appointment slots<br>Use EHR dashboards to tell real time capacity across hospital<br>Use heat maps to see pressure over the week<br>Use heat maps to see where the highest work load is as base for improvements
	Use some type of patient coordinators to see and prioritize the needs and process of the patient	A nurse patient coordinator has global picture of each department and takes decisions<br>A patient navigator to traffic patients visits and book surgery<br>Command center physician leader and admitting services works close to create smooth transition from ED to ward<br>Flow coordinators to identify reoccuring problems<br>Have multiple groups represented in command centers<br>Have the same coordinator for returning patients<br>Nurses as operating managers in charge of patient flow<br>Nurses in expediting flow role at command center<br>Patient flow speciliasts coordinates flow<br>Patient navigators needs to have access to all schedules, know patients situation and have access to the EHR<br>Physician or nurse as patient flow officer in command center<br>Senior nurses to coordinate use of bed capacity
	Work with closer collaboration between clinics or departments across the hospital	Clinics help each other and move staff between them if someone is sick<br>Complex patients stays at designated ward, simpler patients can be transferred to other wards<br>Cooperate across clinics and identify need of staff<br>Department groupings for shared patient care<br>Good coordination between management groups of the OR, the ICU and the surgical wards<br>Identify partner units to pool capacity<br>If clinic is full then other clinics give beds to patient in joint coordination<br>If overcrowded then keep patients in the PACU to lower pressure on wards<br>It is extra important to coordinate the ED, the ICU and the OR<br>Make wards used to different patients to hospitalize patients in other departments<br>Retake patients to their proper dept. after situational relocation<br>Use the recovery room to create capacity if their is space and availability<br>When the ward is full then find beds in other specialities as long as its safe<br>When ward is full, patients can get a bed at other wards as long as its medically safe

Themes	Solution Categories	Open Interview Codes
Transfer	Give specific flow unit or team the task to control and arrange for efficient transfers	A coordinating unit can expediate ED transfer when no clinic wants the patient
		Care practice consultants to coordinate transfer between outpatient practices and the hospital
		Flow department control beds and the flow of patients
		Flow department moves patients between services to optimize flow and care
		Flow team has mandate to move a patient to other ward if there is a free bed
		Have flow coordinators has strong mandate to optimise the flow of patients
		Have flow coordinators with strong mandate to optimize flow
		If clinic can't solve problem locally then flow team finds solution across hospital
		Internal efficiency by cooperation between respective physician and coordinator
		Main task of flow team is to get patients out of the ED in 10 hours
		Patient flow department controls and optimizes use of beds
		Patient placement department work with flow, admissions, discharge and transfer
		The patient flow deparment controls bedflow and everything must go through there
		Use flow coordinators with strong mandate to optimise the flow of patients
	Have clear roles with defined mandates concerning transfers between the ED and the receiving clinic	An internal transport center is responsible for transfer
		ED decides patient transfer and is responsible until delivery
		Emerency department identifies the receiving service that then must bring the patient from the ED
		Improve transfers to not only rely on the management of relationships
		Physicians admits but nurses control the flow from the ED to services
		Receiving service cannot object to patient being sent to them
		The ED has mandate to decide where patients are sent
		To expediate ED transfer each department has a reference physician at the ED
		Triage physicians dictates ward placement flexibility for patient
		Use a pull system to make the wards pull a patient up from the ED when a bed is ready
	Have standardized handoffs, pre-defined destinations and established incentives for efficient transfers	Create clear view of where ICU patients can be transferred when stabilized
		Have clear standards and handoff routines between outpatient and inpatient care
		Have standardized templates for handoffs built into the electronic health record
		Make transfer handoffs correct and efficient
		Must be a quick doctor-doctor handoff for efficient transfers
		Patient transfers depend on good personal communication
		Use a detailed admission document where algorithm decides service of transfer to avoid any discussion
		Use of frequency list to expediate transfer from ED to services
	Use digital tools to efficiently connect, direct and navigate cleaners, porters and ambulatory patients	Create an efficient system on how to notify cleaners to clean empty beds
		Create the right incentives to transfer patients from the ICU
		Have an efficient IT-system for patient transfers with connected porters
		Have an efficient website for patient navigation
		Provide digital time travelling applications to all patients to expediate transfers
		Use more cleaningstaff to quickly enable beds before transfer
