# Supplementary material for: Solutions for improved hospital-wide patient flows – a qualitative interview study of leading healthcare providers
Source: BMC Health Serv Res. 2023 Jan 7;23:17. doi: 10.1186/s12913-022-09015-w (PMC9825009; doi:10.1186/s12913-022-09015-w)
Supplement: Supplementary file 3 — Additional file 3: Appendix C. The number of hospitals that acknowledges the need to work with a particular solution is categorized from the highest to the lowest number together with the name of the solution and associated theme. [file 12913_2022_9015_MOESM3_ESM.pdf]

Appendix C:

The number of hospitals that acknowledges the need to work with a particular solution is categorized from the highest to the lowest number together with the name of the solution and associated theme.

Themes	Solutions	Hospitals/ solution
Healthcare sector	Increase staffing and bed capacity across the whole healthcare system	**14**
Discharge	Enable discharge predictability with more home care solutions and own downstream facilities	**14**
Discharge	Share objectives, information and real time capacity data with after care services	**13**
Internal	Give clinics trust and improvement autonomy but follow central process metrics and external benchmark	**11**
Management System	Use a flow command center to optimize capacity use and break hospital flow bottlenecks	**11**
Management System	Use IT-tools to analyze bed capacity use and provide daily real time visibility on hospital capacity	**11**
Discharge	Have dedicated discharge coordinators or coordinating teams	**11**
Discharge	Prioritize activities and organize staff to ensure early and efficient daily discharges	**10**
Discharge	Set early discharge goals and work towards them along the patients' whole hospital journey	**10**
Entry	Require increased primary care responsibility and support with more knowledge exchange and coordination	**10**
Entry	Strategic planning: make recurring strategic revisions on fit between demand and capacity	**10**
Entry	Strategic planning: use predictive analytics to forecast demand patterns and capacity needs	**10**
Internal	Improve prioritization schemes and develop standards on procedures, roles and staff-bed ratios	**10**
Internal	Optimize and smooth occupancy rate levels by admitting patients based on length of stay and ICU risk	**10**
Internal	Understand the tipping point of hospitals' capacity utilization and ensure sufficient capacity buffers	**10**
Internal	Use an OR block schedule per clinic and plan cases based on downstream bed availability	**10**
Management System	Build up flexible hospital-wide capacity to handle peaks or capacity unbalances	**10**
Management System	Ensure sufficient capacity along the whole patient flow when setting objectives to avoid bottlenecks	**10**
Management System	Use some type of patient coordinators to see and prioritize the needs and process of the patient	**10**
Transfer	Give specific flow unit or team the task to control and arrange for efficient transfers	**10**
Entry	Reach, inform and treat patients before they seek acute hospital care	**10**
Internal	Ensure a high OR-utilization with smart case mixes, all day utilization and quick cancellation refill	**9**
Internal	Use more digital tools and new treatment methods to reduce lead times	**9**
Internal	Utilize as much of the week as possible and staff day and week according to real demand patterns	**9**
Management System	Connect managers and staff across the hospital to break silo mindsets	**9**
Management System	Put patient flow focus on top of the agenda across the hospital, to change the culture	**9**
Discharge	Request and work towards increased responsibility from after care services	**8**
Discharge	Use mutual staffing collaboration between the hospital and after care services	**8**
Entry	Cooperate with other hospitals to ensure bed capacity and to seek appropriate level of care	**8**
Entry	Use IT-tools and data-analysis for standardized admissions, early assessments and reduced no-shows	**8**
Internal	Schedule staff and all clinical activities based on an optimal utilization of the OR-schedule	**8**
Management System	Work with closer collaboration between clinics or departments across the hospital	**8**
Transfer	Have standardized handoffs, pre-defined destinations and established incentives for efficient transfers	**8**
Discharge	Increase prioritization of discharge ready patients when planning procedures and activities	**8**
Discharge	Provide follow up appointments at discharge to ensure accountability and continuity	**7**
Entry	Ensure capability to reroute less severe ED patients to outpatient or ambulatory care	**7**
Internal	Have a structured organization for daily problem solving and capacity optimization	**7**
Management System	Operational planning: have daily capacity meetings with all clinics or departments of the hospital	**7**
Transfer	Have clear roles with defined mandates concerning transfers between the ED and the receiving clinic	**7**
Transfer	Use digital tools to efficiently connect, direct and navigate cleaners, porters and ambulatory patients	**7**
Internal	Make all employees understand the importance of having a patient flow focus	**7**
Internal	Operational planning: have daily capacity meetings within the department or clinic	**7**
Management System	Align objectives, metrics and patient data systems (EHR & CRM) across the organization	**6**
Healthcare sector	Create healthcare system alignment with clear goals and objectives for each healthcare actor	**6**
Internal	Improve outpatient processes by implementing standards on schedules and appointments	**6**
Management System	Share and visualize correct data across the organization to make everyone understand flow implications	**5**
Management System	Tactical planning: have weekly capacity coordination meetings with all clinics of the hospital	**5**
Internal	Allocate dedicated capacity for both acute and elective patient flows	**4**
Internal	Invest in ancillary service capabilites to minimize bottleneck risks in indirect patient activities	**4**
Internal	Use external facilities or patient hotels to release hospital bed capacity	**4**
